# The hydrogen peroxide-sensitive proteome of the chloroplast *in vitro *and *in vivo*

**DOI:** 10.3389/fpls.2013.00054

**Published:** 2013-03-19

**Authors:** Meenakumari Muthuramalingam, Andrea Matros, Renate Scheibe, Hans-Peter Mock, Karl-Josef Dietz

**Affiliations:** ^1^Biochemistry and Physiology of Plants, Faculty of Biology – W5-134, Bielefeld UniversityBielefeld, Germany; ^2^Applied Biochemistry, Institute of Plant Genetics and Crop Plant ResearchGatersleben, Germany; ^3^Plant Physiology, Faculty of Biology and Chemistry, University of OsnabrückOsnabrück, Germany

**Keywords:** chloroplast proteome, hydrogen peroxide, methyl viologen, ribulose-bisphosphate carboxylase, redox regulation

## Abstract

Hydrogen peroxide (H_2_O_2_) evolves during cellular metabolism and accumulates under various stresses causing serious redox imbalances. Many proteomics studies aiming to identify proteins sensitive to H_2_O_2_ used concentrations that were above the physiological range. Here the chloroplast proteins were subjected to partial oxidation by exogenous addition of H_2_O_2_ equivalent to 10% of available protein thiols which allowed for the identification of the primary targets of oxidation. The chosen redox proteomic approach employed differential labeling of non-oxidized and oxidized thiols using sequential alkylation with *N*-ethylmaleimide and biotin maleimide. The *in vitro* identified proteins are involved in carbohydrate metabolism, photosynthesis, redox homeostasis, and nitrogen assimilation. By using methyl viologen that induces oxidative stress* in vivo*, mostly the same primary targets of oxidation were identified and several oxidation sites were annotated. Ribulose-1,5-bisphosphate (RubisCO) was a primary oxidation target. Due to its high abundance, RubisCO is suggested to act as a chloroplast redox buffer to maintain a suitable redox state, even in the presence of increased reactive oxygen species release. 2-cysteine peroxiredoxins (2-Cys Prx) undergo redox-dependent modifications and play important roles in antioxidant defense and signaling. The identification of 2-Cys Prx was expected based on its high affinity to H_2_O_2_ and is considered as a proof of concept for the approach. Targets of Trx, such as phosphoribulokinase, glyceraldehyde-3-phosphate dehydrogenase, transketolase, and sedoheptulose-1,7-bisphosphatase have at least one regulatory disulfide bridge which supports the conclusion that the identified proteins undergo reversible thiol oxidation. In conclusion, the presented approach enabled the identification of early targets of H_2_O_2_ oxidation within the cellular proteome under physiological experimental conditions.

## INTRODUCTION

Chloroplasts are essential organelles in plant cells with a wide range of metabolic functions. The redox cascades of the light-driven photosynthetic electron transport chain provide the driving force for metabolism, but they also conditionally generate oxidizing power in the form of reactive oxygen species (ROS). ROS levels increase due to several environmental factors influencing the photosynthetic efficiency, which in turn changes the redox state of the plastid (Foyer and Noctor, 2003). The redox state is also instrumental in regulating the chloroplast metabolic activities but also plastid and nuclear gene expression (Allen, 2003; Pfannschmidt, 2003; Pogson et al., 2008). Thus, chloroplasts serve as an excellent model for better understanding of the redox system which enhances the plant tolerance to environmental stresses. Because of their central role in plant cell signaling, chloroplasts are also considered to function as sensors of environmental fluctuations. According to this scenario, the redox status of chloroplasts is crucial in biological stress response and helps the plant to cope with environmental changes (Scheibe and Dietz, 2012).

Cysteine (Cys) residues in proteins harbor thiol side chains that are highly reactive toward oxidants and can undergo various redox-based modifications. The oxidation of sensitive Cys may cause intra- or intermolecular disulfides. The concomitant conformational changes often regulate the activity and protect the critical thiols against irreversible oxidation (Brandes et al., 2009; König et al., 2012). Reactive thiols can form higher oxidation states like sulfenic acid (SOH) and sulfinic acid (SO_2_H), which are reversed by thiol-specific cellular reductants like glutathione, thioredoxin, or sulfiredoxin (Liu et al., 2006). Hyperoxidation describes two forms of Cys oxidation, namely the SO_2_H and sulfonic acid (SO_3_H) states, the latter one being irreversible to our present-day knowledge. Cys can also form mixed-disulfides with glutathione (glutathionylation) or is *S*-nitrosylated. Both modifications receive increasing attention as important redox regulatory mechanism in biology (Mieyal and Chock, 2012). Through these different post-translational modifications Cys appears to be involved in virtually all cellular activities, including immediate metabolic regulation and control of transcriptional and translational activities in development and defense.

Analytical methods have been developed to detect reversible thiol oxidation and they employ combinations of labeling and blocking strategies. The challenge is to identify a few oxidized disulfides among the mass of reduced thiols of a healthy cell. Generally this method includes a saturating blockage of free thiols with thiol reactive reagents, followed by reduction of the disulfides (Muthuramalingam et al., 2010). Subsequently, the newly exposed thiol groups from the reduction step are labeled with detectable thiol-specific reagents. Depending on the specificity of the thiol reductant, this method can be used to identify all reversible thiol modifications (Leichert and Jakob, 2004). Novel labeling techniques based on isotope-coded affinity tags (ICAT) enable quantification of differential protein expression (Sethuraman et al., 2004). The combination of labeling strategies and advanced proteomic methodologies led to the identification of redox proteins which are regulated by thiol-disulfide transitions (Motohashi et al., 2001; Buchanan and Balmer, 2005; Rouhier et al., 2005; Bartsch et al., 2008; Ströher and Dietz, 2008). Often these studies employ rather extreme oxidizing condition. Thus a major open issue concerns the question as to which of the redox-regulated proteins are the primary targets of oxidation and how an initial redox imbalance is sensed in the cells.

Hydrogen peroxide (H_2_O_2_) is a by-product of normal metabolism and has a sufficient half-life to allow its spreading throughout the entire cell (Bhattachrjee, 2005). H_2_O_2_ is involved in a number of signaling cascades (Neill et al., 2002) and also in programmed cell death in plants (Levine et al., 1994). A recent study has shown that aquaporins facilitate the movement of H_2_O_2_ across the membrane (Bienert et al., 2007). As thiols play major roles in ROS-mediated signaling pathways, identification of thiols that are most sensitive to H_2_O_2_ will help to understand the redox-signaling pathways. Several proteomics studies have addressed the effects of H_2_O_2_ treatment of seedlings, roots and shoot on proteome composition and carbonylation state of proteins (Tanou et al., 2010; Barba-Espín et al., 2011; Zhou et al., 2011). These studies provide important insight into non-redox effects of H_2_O_2_ stress and downstream events of H_2_O_2_-dependent signaling in plants.

In this context, the present study focuses on the identification of chloroplast stroma proteins which are most sensitive to H_2_O_2_. *Arabidopsis thaliana* stroma proteins were subjected to partial oxidation by exogenous addition of limited amounts of H_2_O_2_ in order to observe the global response of the chloroplast redox network to an oxidizing stimulus. Initial targets of oxidation were identified by mass spectrometry (MS). In order to confirm the response of identified proteins to oxidation *in vivo*, plants were subjected to methyl viologen (MV) treatment. MV is a redox-active herbicide that accepts electrons at the photosystem I site and produces superoxide through reduction of oxygen within the chloroplasts (Halliwell and Gutteridge, 1989; Jacob and Dietz, 2009). The approach was successfully established and a first list of ROS-sensitive stroma proteins could be provided. Surprisingly, ribulose-1,5-bisphosphate carboxylase oxygenase (RubisCO) proved to be a prominent target *in vitro* and *in vivo*, allowing us to hypothesize on its redox-buffering function during episodes of transient oxidative stress.

## MATERIALS AND METHODS

### GROWTH OF *ARABIDOPSIS THALIANA* AND CHLOROPLAST ISOLATION

**FIGURE 1:**
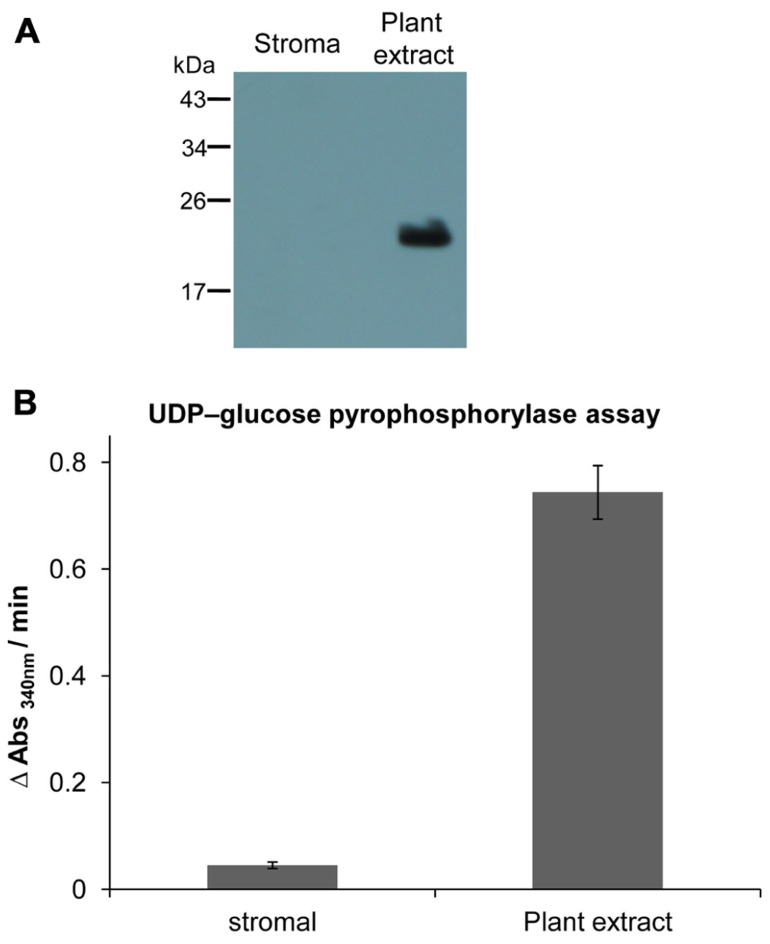
**Estimation of cross-contamination of purified chloroplast fraction**. **(A)** Western blot analysis of stroma and plant extract proteins using specific antibody against AtPrxII F (Finkemeier et al., 2005). Equal amounts of protein (25 μg) were loaded in each lane. AtPrxII F was detected as monomer around 21 kDa and was only found in total leaf extract. **(B) **Activity of UGPase as cytosolic marker enzyme, represented as rate of NADPH+H^+^ formation in plant extract and stroma fraction. The assay was performed three times. Data express means ± SD.

*Arabidopsis thaliana* (ecotype Columbia) was grown in soil culture with 10 h light/14 h darkness at 22/18°C, respectively, and a photosynthetic photon fluence rate of 120 μmol quanta m^-2^ s^-1^. 6 week old plants were used for the chloroplast isolation. Leaves were harvested and homogenized in buffer containing 0.3 M sorbitol, 20 mM Tricine/KOH (pH 8.4), 5 mM ethylenediaminetetraacetic acid (EDTA) and 2 mM ascorbic acid. The homogenate was filtered through eight layers of muslin cloth and nylon mesh. The debris was removed by centrifugation at 3000 rpm and 4°C for 2 min. The sedimented chloroplasts were resuspended in isolation buffer, containing 0.33 M sorbitol, 5 mM MgCl_2_, 20 mM HEPES/KOH (pH 7.9), 2 mM EDTA with freshly added ascorbate. The resuspended chloroplasts were loaded on top of a Percoll step gradient consisting of layers with 40 and 80% Percoll medium containing 0.02 g Ficoll and 0.1 g PEG. The gradient was centrifuged at 3000 rpm for 30 min without brakes. Intact chloroplasts were collected from the interphase between the Percoll layers and washed twice by spinning at 3000 rpm for 2 min. The stroma proteins were extracted following lysis and RubisCO was partially removed according to Ströher and Dietz (2008). The purity of stromal protein preparation was verified by using organelle specific enzymatic and antibody assay (**Figure 1**). The cytosolic marker enzyme UDP-glucose pyrophosphorylase (UGPase) activity was measured according to Zrenner et al. (1993). Using UDP-glucose and pyrophosphate as substrates, Glc-1-P released by UGPase was converted to glucose-6-phosphate (Glc-6-P) which was quantified by coupling to NADP^+^ reduction by Glc-6-P dehydrogenase. Mitochondrial type II peroxiredoxin F (AtPrxIIF) was used as a marker for mitochondrial contaminations. Equal amounts of total plant and stromal proteins (25 μg) were loaded and separated on reducing SDS-PAGE gels. Western blot analysis with antibodies raised against heterologous expressed AtPrxII F was performed as described in Finkemeier et al. (2005).

### DTNB-BASED QUANTIFICATION OF THIOL GROUPS

Total sulfhydryl contents of stroma proteins were determined as described by Tietze (1969). Proteins were precipitated in 3% trichloroacetic acid (TCA) and recovered after a brief centrifugation. To expose buried thiol groups the resulting pellet was dissolved in denaturing buffer containing 100 mM Tris-HCl (pH 8.0), 6 M guanidinium HCl or 1% SDS. The free thiol groups were quantified spectrophotometrically at 412 nm using 6 mM 5,5-dithio-bis(2-nitrobenzoic acid; DTNB) as substrate.

### SAMPLE PREPARATION (*IN VITRO* AND *IN VIVO* OXIDATION TREATMENT)

Partial oxidation *in vitro* was performed in 20 mM Tris-HCl (pH 7.8) buffer by adding H_2_O_2_ in varying stoichiometric quantities equivalent to 1, 2.5, 5, and 10% of the protein thiol content determined by DTNB assay in the respective fraction. The range of ratios which was usually equivalent to 1–10 μM concentrations was selected to identify preferred sites for oxidation. For *in vivo* oxidation, 50 μM MV supplemented with 0.1% Tween-20 was sprayed on whole plants that were harvested after 10, 30 and 60 min. Three independent experiments were performed for both *in vitro* and *in vivo* oxidation treatments and the identified target proteins are representative of respective replicate experiments.

### DETECTION OF REDOX-REGULATED PROTEINS VIA A SEQUENTIAL LABELING STRATEGY

All buffers were depleted from dissolved O_2_ by bubbling with argon gas at room temperature (RT). The stroma protein fraction was completely reduced in the presence of 25 mM dithiothreitol (DTT). The reaction was performed inside a closed microaerobiosis chamber continuously flushed with nitrogen gas produced in a nitrogen generator to maintain the oxygen content at less than 0.4%. Excess DTT was removed by desalting using 10 ml desalting columns. The proteins in 20 mM Tris-HCl (pH 7.8) buffer with about 100 μM thiols were treated with H_2_O_2_(about 10 μM) for 5 min with gentle shaking to identify the initial targets of oxidation. Remaining cysteinyl thiols were alkylated using 100 mM *N*-ethylmaleimide (NEM) in darkness for 1 h to prevent oxidation. Excess NEM was removed by TCA precipitation according to Muthuramalingam et al. (2010). The washed precipitate was solubilized in denaturing buffer containing 200 mM Bis-Tris (pH 6.5), 6 M urea, 0.5% (w/v) SDS and 10 mM EDTA supplemented with 100 mM DTT to allow full reduction of oxidized thiols. Excess DTT was removed by repeated TCA precipitation. The labeling of the rereduced, previously oxidized thiols was achieved with 25 mM biotin maleimide in the dark for 90 min under constant shaking. Excess labeling reagent was removed either by TCA precipitation or with PD-10 desalting columns (GE Healthcare) depending on the downstream processing.

To monitor the *in vivo* redox status of proteins, proteins from MV-treated plants were extracted in the presence of 100 mM NEM. Furtheron, the proteins were reduced with DTT and subsequently labeled with biotin maleimide as described above. Biotinylated proteins were separated either by one or two-dimensional gel electrophoresis (2DE) and subsequently transferred onto nitrocellulose membrane using the semidry blotter Fastblot B44 (Whatman/Biometra, Germany). After a blocking step with 1% fish gelatin (Sigma, Germany) for 2 h at RT, the membrane was probed with anti-biotin antibody (Clone BN-34 from Sigma–Aldrich, St. Louis, USA). Then the membrane was incubated for 1 h with horseradish peroxidase-conjugated anti-mouse antibody (Sigma–Aldrich, St. Louis, USA) and developed using the enhanced chemiluminescence method (Thermo Scientific, Germany).

### STREPTAVIDIN AFFINITY PURIFICATION

The protein samples were desalted to remove labeling solution. This was needed since denaturing reagents used for solubilizing TCA-precipitated protein pellets inhibited binding of biotinylated polypeptides to the streptavidin column. Biotinylated proteins were enriched using streptavidin agarose. The biotinylated sample was incubated with streptavidin agarose equilibrated with phosphate buffered saline (PBS) buffer, pH 7.4, at 4°C with constant shaking overnight. Nonspecifically bound proteins were washed with 1× PBS until the absorbance at 280 nm reached zero. Proteins were incubated with elution buffer containing 1% SDS, 30 mM biotin (pH 12) for 15 min at RT, followed by heating at 96 °C for 15 min.

### IDENTIFICATION OF PROTEINS USING MALDI MS ANALYSIS

Proteins purified by streptavidin agarose were resolved by one-dimensional SDS-PAGE and stained with silver nitrate according to Blum et al. (1987). Spots of interest were excised from the gel and placed in U-shaped microtiter plate wells (Greiner Bio-one, Germany). To remove the silver, the excised gel spots were de-stained using Farmer’s reducing reagent containing 30 mM potassium ferricyanide (III) and 100 mM sodium thiosulfate. Then the gel spots were washed several times with ultrapure H_2_O until the gel slices became transparent. Protein spots were washed twice with 30% (v/v) acetonitrile in 0.1 M ammonium hydrogen carbonate and subsequently dried in the speedvac. The gel pieces were rehydrated in the presence of 0.01 μg trypsin/μl (Promega, Mannheim, Germany) at RT for 30 min followed by overnight incubation at 37°C according to manufacturer’s protocol. The gel slices were vacuum dried, and the peptides were extracted with 50% acetonitrile and 0.1% trifluoroacetic acid for MS analysis. Acquisition of peptide mass fingerprint data and corresponding LIFT spectra was performed using an ultrafleXtreme matrix-assisted laser desorption/ionization time-of-flight (MALDI-TOF) device (Bruker Daltonics, Bremen, Germany) equipped with a Smartbeam-II laser with a repetition rate of 1000 Hz. The spectra were calibrated using external calibration and subsequent internal mass correction. For databank searching, Biotools 3.2 software (Bruker Daltonics) with the implemented MASCOT search engine (Matrix Science) was used, searching for *A. thaliana* in the non-redundant National Center for Biotechnology Information database (26/07/2010, 55602 sequences). Search parameters were as follows: monoisotopic mass accuracy; 50 ppm tolerance; fragment tolerance of 0.3 Da; missed cleavages 1; and the allowed variable modifications were oxidation (Met), propionamide (Cys), and carbamidomethyl (Cys). Proteins were identified from all three independent experiments applying MASCOT significance scores of 60 (protein level) and 32 (peptide level). Proteins found in just one of the experiments are indicated in table legends (**Tables 1–3**).

**Table 1:** *Arabidopsis thaliana* stroma proteins containing H_**2**_O_**2**_sensitive thiols identified by MALDI-TOF/MS.

No.	Protein	Accession number	MM (kDa)	Cys number	Functional role
1	Fd-GOGAT	AT5G04140	165.3	24	Nitrogen assimilation
2	Transketolase	AT3G60750	73.1	8	Photosynthesis
3	RubisCO large subunit	ATCG00490	52.9	9	Photosynthesis
4	Fructose-bisphosphate aldolase-2	AT4G38970	37.9	2	Photosynthesis
5	GAPDH subunit B	AT1G42970	39.3	7	Photosynthesis
6	GAPDH subunit A-2	AT1G12900	37.7	5	Photosynthesis
7	Ferredoxin-NADP(+) oxidoreductase 1	AT5G66190	35.2	5	Photosynthesis
8	Carbonic anhydrase 1	AT3G01500	25.6	6	Photosynthesis
9	2-Cys peroxiredoxin	AT3G11630	22.4	2	Detoxification
10	Cyclophilin 20-3	AT3G62030	19.9	4	Protein folding
11	RubisCO small subunit	AT1G67090	14.7	4	Photosynthesis
12*	Phosphoribulokinase	AT1G32060	39.2	4	Photosynthesis
13*	STN7 kinase	AT1G68830	58.5	5	Serine/threonine kinase
14*	Glutamine synthetase 2	AT5G35630	42.5	6	Ammonia assimilation cycle
15*	ATP synthase beta subunit	ATCG00480	52.5	1	ATP synthesis
16*	HCF 136	AT5G23120	35.8	0	PSII stability complex
17*	DRT 112	AT1G20340	10.5	1	Electron carrier

Each protein is annotated by its name, accession number, molecular weight (without predicted transit peptide), and the biological function. The number of cysteines theoretically present in the mature protein is given. The table compiles the results from three independent experiments. Proteins 1–11 were identified in each experiment. Asterisk “*” denotes that these proteins were identified in single experiments where 10% molar equivalence to protein thiols corresponded to 15 μM H_2_O_2_ concentration.

### BIOTIN QUANTIFICATION ASSAY

The extent of biotinylation was quantified using the HABA-avidin assay developed by Green (1975). HABA forms a red complex with avidin that can be monitored spectrophotometrically at 500 nm. Due to its higher affinity, biotin displaces HABA, accompanied by the decrease in absorbance at 500 nm. The biotinylated protein samples were desalted to remove the excess of biotin maleimide reagent before performing the assay. The assay mixture consisted of 1× PBS buffer containing HABA-avidin reagent (Sigma, Germany) and 10 μg of biotinylated protein sample. After 2 min incubation the absorbance at 500 nm was recorded. The change in absorbance at 500 nm is proportional to the amount of biotin in the assay. A standard curve was generated using free biotin and used to estimate the number of moles of biotin incorporated after biotinylating the protein.

## RESULTS

### PURITY OF THE CHLOROPLAST FRACTION

Chloroplasts were isolated and lysed to obtain a stromal protein fraction which was checked for contaminations by other cellular constituents. Type II peroxiredoxin F (AtPrxII F) was used as a marker for mitochondrial contamination using Western blot analysis. In total plant protein extract AtPrxII F was detected at the expected size of about 21 kDa, while it was absent in the stromal fraction (**Figure 1A**). As shown in **Figure 1B** the plant protein extract exhibited high rates of nicotinamide adenine dinucleotide phosphate (NADPH) formation at 340 nm, representative for high UGPase activity, while its enzymatic activity in the stromal fraction was minimal with less than 6% relative contamination.

### TOTAL PROTEIN THIOL DETERMINATION

The present study aimed to identify the primary protein targets of H_2_O_2_ oxidation in the chloroplast *in vitro* and *in vivo*. Percoll-purified intact chloroplasts were lysed, and the stroma protein fraction was recovered by centrifugation. Total thiol contents of stroma protein extract was determined using the DTNB assay in order to adjust the amount of H_2_O_2_ to be added for oxidation to 10% of total protein thiols. Protein thiols were quantified under reducing and denaturing conditions to obtain an average amount of thiols to be used as a conversion factor for future experiments. Low molecular weight thiol metabolites such as glutathione were removed by TCA precipitation, followed by a centrifugation. Both denaturing methods, namely guanidinium hydrochloride- and SDS-treatment gave a highly similar result of 57.1 ± 4.3 and 58.3 ± 3.1 μmol/g protein, respectively (mean ± SD of *n* = 3), corresponding to an average of 3 Cys per 50 kDa protein.

**FIGURE 2:**
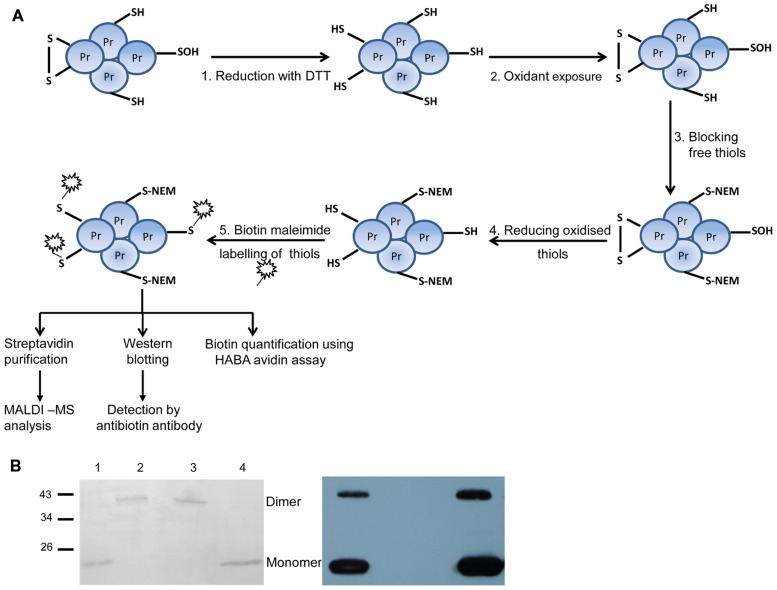
**Overview of the procedure for labeling reversibly oxidized cysteines and test experiment with 2-Cys Prx**. **(A)** Work flow used for identifying protein targets sensitive to low H_2_O_2_ concentrations consisted of five steps: step 1 includes complete reduction of protein thiols using the artificial reductant DTT. Step 2: After desalting to remove excess DTT, proteins were exposed to H_2_O_2_ for 5 min. In step 3, free thiol groups were blocked using NEM under denaturing conditions. Reversibly oxidized thiols were reduced with DTT in step 4. The newly exposed thiol groups were labeled using the thiol specific reagent biotin maleimide in step 5. As a control reaction, proteins were not subjected to oxidation (step 2) while all other steps were done as described above. From steps 1 to 3, the samples were treated inside the microaerobic chamber with <0.4% O_2_. Subsequent steps were performed in ambient air. **(B)** Test of experimental workflow with 2-Cys Prx. Each subunit of the 2-Cys Prx dimer has two cysteines. Biotin labeling of reduced 2-Cys Prx (lane 1); oxidized (lane 2); reduced and blocked with NEM (lane 3), and reduced, oxidized and re-reduced sample (lane 4). On the left is the Ponceau S-stained membrane which shows equal protein amounts loaded. The corresponding blot developed with antibody against biotin is shown on the right and is representative for three experiments.

In order to optimize the workflow (**Figure 2A**) and to check for reaction specificity, 2-cysteine peroxiredoxin (2-Cys Prx) was used as test protein, since it is a well characterized redox-regulated protein (**Figure 2B**). 2-Cys Prx has two cysteinyl residues and forms an intermolecular disulfide bond upon oxidation. Thus the oxidized form runs as a dimer on non-reducing SDS-PAGE. To check the specificity of the labeling strategy, proteins were directly labeled with biotin maleimide after each step of the work flow and detected immunologically with antibody against biotin. As shown in **Figure 2B**, free thiols were not available for biotin maleimide labeling after oxidation of 2-Cys Prx as indicated by the absence of signal in the Western blot (lane 2). A similar result was observed after blocking of free thiols with NEM, an alkylating agent to prevent thiol-disulfide exchange reactions (lane 3). After reduction, Cys were efficiently labeled with biotin maleimide and strong bands were detected in the blot (lane 1 and 4). The band detected around 48 kDa corresponds to the half-oxidized dimer, since each dimer contains two catalytic sites each of which can form a disulfide bridge.

### EFFECT OF H_2_O_2_-MEDIATED OXIDATION ON STROMA THIOL PROTEINS

**FIGURE 3:**
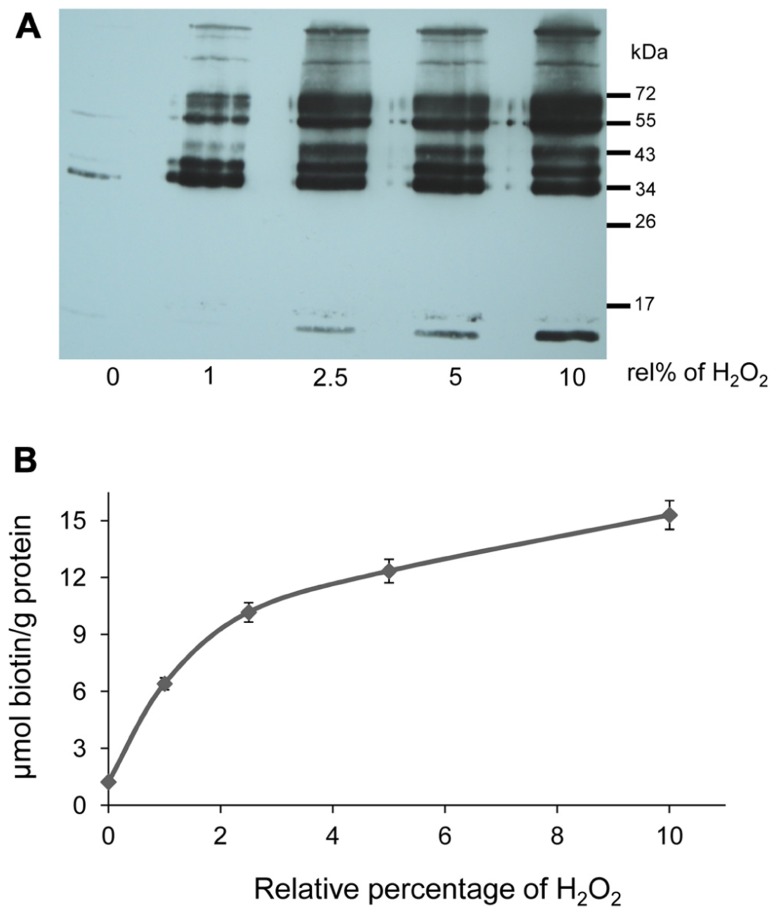
**H_2_O_2_-dependent oxidation of stroma proteins**. **(A)** Western blot analysis with anti-biotin antibody to detect oxidized stroma proteins. “rel%” stands for the amount of H_2_O_2_ added relative to the thiol contents of the protein sample (100%). The bands demonstrate H_2_O_2_-dependent oxidation of stroma proteins. Same samples were used for biotin quantification. The experiment with similar results was performed three times. **(B)** Degree of biotinylation in H_2_O_2_-treated samples as calculated from HABA displacement assay. For biotin quantification, 10 μg of proteins were used. Data are means ± SD, *n* = 3.

Stroma proteins were subjected to H_2_O_2_ oxidation, subsequently reduced and labeled with biotin maleimide. Oxidation was performed by adding H_2_O_2 _ at amounts of varying stoichiometry relative to protein thiol contents (1, 2.5, 5, and 10%) which corresponded to 1 to 10 μM concentration. Under optimal conditions for photosynthesis, H_2_O_2_ concentrations are considered to be below 1 μM. H_2_O_2_ accumulates under stress. 10 μM H_2_O_2_ inhibits the Calvin cycle in isolated chloroplast by half (Kaiser, 1976; Asada, 1999; Polle, 2001). Remaining free thiols were blocked with NEM followed by reduction of reversibly oxidized proteins with DTT. The newly recovered thiol groups were then labeled with biotin maleimide. Hence biotinylated proteins corresponded to Cys-containing proteins that had been reversibly oxidized by the added H_2_O_2_. The increase in biotin label corresponding to increased oxidation is shown in **Figure 3A**. In the control reaction, the proteins were reduced and directly blocked with NEM without exposure to H_2_O_2_. Complete reduction and immediate blocking of free thiols in the control sample resulted in only minor incorporation of biotin maleimide into proteins (**Figure 3A**; lane 1). The labeling degree increased with increasing H_2_O_2_ concentrations.

The amount of biotin maleimide in the labeled samples was quantified with the HABA-avidin assay. Equal amounts of protein from different H_2_O_2_-treated and control samples were mixed with HABA-avidin reagent. The assay displayed a decrease in the absorbance that is proportional to the amount of biotin maleimide present in the sample. The degree of biotinylation was calculated as micromol biotin-labeled thiol per gram protein. As expected the increasing amounts of H_2_O_2_ and subsequent reduction allowed the incorporation of more biotin, representing the extent of thiol oxidation (**Figure 3B**).

**FIGURE 4:**
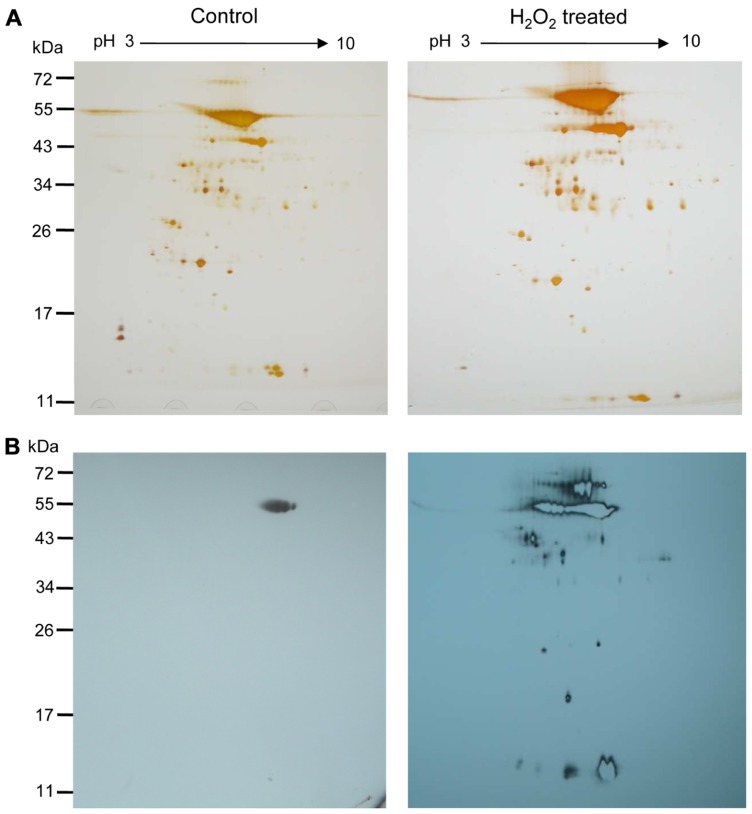
**H_2_O_2_-mediated oxidation of stroma proteins**. **(A)** Two-dimensional map of the *A. thaliana *stroma proteins after differential labeling. Separation was performed on 18 cm Immobiline DryStrip (pH 3–10 NL) and by 12% SDS-PAGE as second-dimension. Silver-stained gels of stroma proteins (100 μg) that remained untreated (left) and were treated with H_2_O_2_ at about 10 μM for 5 min (right) are presented. **(B)** Two-dimensional Western blot map after decoration with anti-biotin antibody. Control and H_2_O_2_-treated samples of the respective gels are shown. Oxidized protein thiols were labeled with biotin maleimide as described. The figure is representative of three independent experiments. Molecular weight markers, in kDa, are indicated on the left.

Two-dimensional gel electrophoresis was performed in order to get insight into the complexity of the H_2_O_2_-mediated oxidative changes. One hundred micrograms of biotin maleimide-labeled control and H_2_O_2_-treated proteins were separated by 2DE and subsequently visualized by silver staining (**Figure 4A**). The corresponding Western blot membranes probed with anti-biotin antibody are shown in **Figure 4B**. The patterns of biotinylated polypeptides differed strongly between control and treated samples, while the patterns from the silver-stained gel detecting total proteins revealed a similar spot pattern despite the fact that apparently slightly more protein had been solubilized in the H_2_O_2_-treated sample. The direct comparison of the blots and the silver-stained gels for identifying and excising the proteins of interest appeared unreliable due to the expected background of unlabeled polypeptides. Therefore the biotinylated proteins were further enriched by purification via streptavidin agarose column chromatography.

### PURIFICATION AND IDENTIFICATION OF BIOTINYLATED PROTEINS FOLLOWING H_2_O_2_-MEDIATED OXIDATION

**FIGURE 5:**
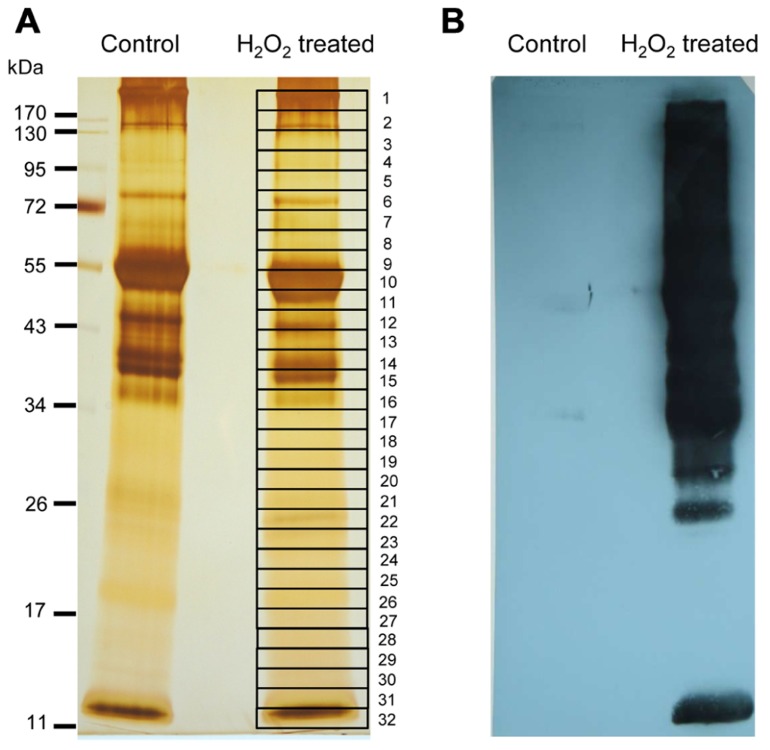
**Identification of early targets of H_2_O_2_-mediated protein oxidation**. **(A)** Silver-stained SDS-PAGE gel showing the biotinylated proteins purified via streptavidin-agarose affinity column. Equal amounts of eluted protein were loaded from control and H_2_O_2_-treated samples. Band numbered on the gel corresponds to the excised gel sections which were trypsin-digested and analyzed by MALDI-TOF MS. Proteins identified with significant MASCOT score >75 are shown in **Table 1**. At least two peptides were identified per protein. **(B)** Western blot visualization of biotinylated proteins with anti-biotin antibody. Equal amounts of protein (100 μg) were loaded in case of the untreated control and H_2_O_2_-treated samples. The result is representative of three independent experiments.

Biotin-labeled control and H_2_O_2_-treated samples were purified by streptavidin agarose chromatography to separate the H_2_O_2_-sensitive biotinylated proteins from the complex protein mixture. Affinity-purified proteins were precipitated with TCA (10% w/v) to remove excess biotin from elution. The protein samples were resolved by one-dimensional SDS-PAGE analysis and visualized by silver staining (**Figure 5A**). Both control and H_2_O_2_-treated proteins exhibited a similar pattern on the gel, which is explained by loading equal protein amounts. However, immuno-reactive signals only appeared on the blot from H_2_O_2_-treated samples, confirming that the biotin labeling was linked to protein oxidation by H_2_O_2_ (**Figure 5B**). To identify proteins containing redox sensitive Cys thiols the indicated gel sections were excised from the gel. Results of MALDI-TOF/MS analysis are summarized in **Table 1**. In total 17 proteins were identified from affinity purified H_2_O_2_-treated samples. All identified proteins are located to the chloroplast. Five proteins function in the Calvin cycle (protein # 2, 5, 6, 11, and 12). The other identified proteins have various functions, such as nitrogen assimilation (proteins #1 and 14), adenosine triphosphate (ATP) synthesis (protein #15) and electron transport (protein # 17) among others. The analysed amino acid composition of these proteins revealed that except for the photosystem II (PSII) stability/assembly factor HCF136 (protein #16) one or more Cys are present in all identified proteins. To confirm oxidation-mediated Cys modification in H_2_O_2_-treated samples, the mass lists of unmatched peptides were compared with the predicted mass of *in silico* trypsin-digested and biotinylated peptides (**Table 2**). This approach allowed us to confirm two peptides of the large subunit (LSU) and small subunit (SSU) of RubisCO, single peptides of ferredoxin-dependent glutamate synthase (Fd-GOGAT), subunit B of GAPDH and ferredoxin-NADP oxidoreductase (FNR).

**Table 2:** Identification of cysteines modified upon oxidation.

Protein identified	Peptide sequence	Predicted mass		Observed mass in H_2_O_2_
		NEM	Biotin-mal	
RubisCO large subunit	AVYEC*LR	978	1304.5	1304.6*
	VALEAC*VQAR	1184.2	1510.7	1510.7*
Fd-GOGAT	IC*NVDR	843.8	1170.3	1170.6*
	FCTGGMSLGAISR	1424.5	1751	1424.7
	ALYYLCEAADDAVR	1697.7	2024.2	1697.8
	IGFVPEEATIVGNTCLYGATGGQIFAR	2910.1	3234.2	2909.5
Ferredoxin-NADP(+) oxidoreductase 1	C*LLNTK	815.9	1142.4	1142.6
	TVSLCVK	873.9	1200.4	873.5
	GVC*SNFLCDLK	1304.5	1631	1630.8*
GAPDH subunit B	GILDVC*DAPLVSVDFR	1843.9	2170.5	2170*
RubisCO small subunit	LPLFGCTDSAQVLK	1616.6	1943.3	1616.9
	QVQC*ISFIAYKPPSFTG	2011.2	2337.7	2337.2*
	WIPC*VEFELEHGFVYR	2149.3	2475.8	2475.2*

Each entry indicates the peptide sequence and the predicted mass in the presence of the alkylating agents either NEM (+125 Da) or biotin maleimide (+451.5 Da). The mass observed in MALDI-TOF/MS of the H_2_O_2_-treated sample is shown. Mass of NEM-labeled Cys matches to experimental mass value suggesting that these Cys are not modified during oxidation. C* marks Cys in the peptide sequence which is preferentially labeled with biotin maleimide, since the predicted mass matches to the measured value. Here, the predicted mass of biotin maleimide-labeled peptide was not found in the corresponding control sample. These data suggest that the approach used can identify proteins sensitive to oxidation and in addition locates the Cys modification.

## PURIFICATION AND IDENTIFICATION OF BIOTINYLATED PROTEINS FOLLOWING MV-MEDIATED OXIDATION

**FIGURE 6:**
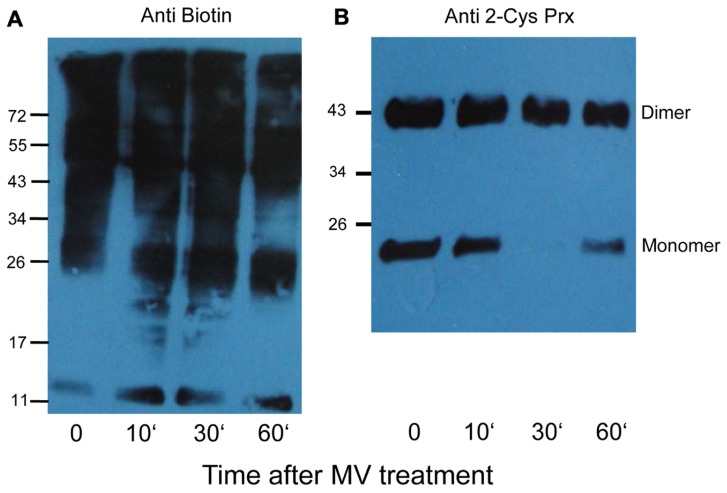
**Biotinylated proteins from *A. thaliana* leaves after methyl viologen treatment**. **(A)** The immunoblot shows increased labeling in protein extracts after treating plants with 50 μM MV for different time periods. Control plants (0) were sprayed with water. All samples were extracted in the presence of NEM to block free thiols, then reduced with DTT, and finally labeled with biotin maleimide. **(B)** The same samples were probed with 2-Cys Prx antibody to determine the redox state of 2-Cys Prx. The oxidized dimer and reduced monomers are indicated.

To determine whether and which proteins are oxidized *in vivo*, 6 week old plants were sprayed with MV that induces photo-oxidative stress. The MV treatment of plants revealed a slight increase in biotinylated proteins after different times (**Figure 6A**). Under oxidizing conditions the catalytic Cys of 2-Cys Prx form an intermolecular disulfide bridge, and it runs as dimer at about 43 kDa, whereas the fully reduced form runs as monomer of 22 kDa. At higher oxidant concentrations 2-Cys Prx is prone to overoxidation, which also results in a monomer (data not shown). After 30 min of MV treatment 2-Cys Prx was fully oxidized as shown in the immunoblot analysis (**Figure 6B**). At the later time points the protein was found as monomer again, suggesting overoxidation. Based on the redox behavior of one of the early target proteins, namely 2-Cys Prx, the 30-min exposure time was selected for further experiments.

After 30 min exposure to MV, proteins were extracted in NEM-containing buffer. Extracted and alkylated proteins were reduced, free thiols labeled with biotin maleimide and subsequently purified via streptavidin column. After elution, the proteins were resolved by SDS-PAGE and identified using MS (**Table 3**). In total 24 proteins were repeatedly identified from affinity purified MV-treated samples. Most identified proteins are located to the chloroplast, while some are located to the cytoplasm (protein #7, 10, 11, 12, and 22), the vacuole (protein #2), the peroxisome (protein #18) and the mitochondrion (protein # 20). Some target proteins such as RubisCO, myrosinase, and fructose-bisphosphate aldolase-2 were found both in control and MV-treated plants. Proteins that were differentially oxidized by MV treatment are discussed below. Among these were 2-Cys Prx, sedoheptulose-1,7-bisphosphatase (SBPase), subunits of the water-oxidizing complex and FNR1. Six *in vivo* identified proteins were common with the *in vitro* H_2_O_2_-treated sample, namely RubisCO LSU and SSU, fructose-bisphosphate aldolase-2, 2-Cys Prx, plastocyanin (DRT 112) as well as FNR. Most identified chloroplast proteins function in photosynthesis (proteins # 8, 9, 14, and 15) and redox homeostasis (proteins # 5, 19, 20, 21, and 24), while others are involved in photorespiration (protein #1), Calvin cycle (protein #6 and 16) and electron transport (proteins #13 and 17).

**Table 3:** Oxidation-susceptible proteins in *A. thaliana* treated with methyl viologen, subjected to differential labeling and identified by MS.

No.	Protein name	Accession number	Molecular weight (kDa)	Number of Cys	Localization	Function
1	RubisCO large subunit	ATCG00490	52.9	9	Chloroplast	Photosynthesis
2	Myrosinase	AT5G26000	61.1	9	Vacuole	Defense
3	Chaperonin 60 subunit alpha 1	AT2G28000	62.1	3	Chloroplast	Chaperonin
4	Fructose-bisphosphate aldolase-2	AT4G38970	43.0	2	Chloroplast	Photosynthesis
5	2-Cys peroxiredoxin	AT3G11630	29.1	2	Chloroplast	Detoxification
6	Sedoheptulose-1,7-bisphosphatase	AT3G55800	42.4	7	Chloroplast	Photosynthesis
7	Annexin D1	AT1G35720	36.2	2	Cytoplasm/Membranes	Detoxification
8	PS II oxygen-evolving complex 1 (PsbO-1)	AT5G66570	35.1	4	Chloroplast	Photosynthesis
9	PS II oxygen-evolving complex 23K protein (PsbP-1)	AT1G06680	28.1	3	Chloroplast	Photosynthesis
10	Glutathione S-transferase F2 (AtGSTF2)	AT4G02520	24.1	0	Cytoplasm	Detoxification
11	Glutathione S-transferase TAU19 (AtGSTU19)	AT1G78380	25.7	1	Cytoplasm	Detoxification
12	Glutathione S-transferase TAU20 (AtGSTU20)	AT1G78370	25.0	2	Cytoplasm	Detoxification
13	Putative plastocyanin (DRT112)	AT1G20340	17.0	1	Chloroplast	Photosynthesis
14	PS II oxygen-evolving enhancer (PSBQ-2)	AT4G05180	24.6	0	Chloroplast	Photosynthesis
15	PS II oxygen-evolving enhancer (PSBQ-1)	AT4G21280	23.9	0	Chloroplast	Photosynthesis
16	RubisCO small subunit	AT1G67090	20.2	5	Chloroplast	Photosynthesis
17	Thioredoxin M1	AT1G03680	19.7	4	Chloroplast	Photosynthesis
18	Putative peroxisomal (S)-2-hydroxy-acid oxidase 2	AT3G14420	40.3	1	Peroxisome	Oxidoreductase
19	NAD(P)-binding Rossmann-fold-containing protein	AT2G37660	34.9	2	Chloroplast	Oxidoreductase
20	Glutathione S-transferase DHAR1	AT1G19570	23.6	2	unclear	Detoxification
21	Ferredoxin-NADP(+) oxidoreductase 1 (FNR1)	AT5G66190	40.3	6	Chloroplast	Photosynthesis
22	Kinesin-5	AT4G05190	89.2	11	Cytoplasm	Microtubule motor
23	Nucleoside diphosphate kinase 2	AT5G63310	25.6	5	Chloroplast	Metabolism
24	Unknown protein (partial)		7.0	4		Probable peroxin

*Each protein is annotated by its name, accession number, molecular weight, subcellular localization, and biological function as given in the plant protein database (PPDB). The number of cysteines theoretically present in the mature protein is given. The table compiles the results from three independent experiments. Proteins 3, 7, 10, 12, 18, 20, 22, 23, and 24 were significantly identified in one experiment only.*

Except for the glutathione S-transferase (GST) F2 (protein #10), PSBQ-2 (protein #14), and PSBQ-1 (protein #15), one or more Cys are theoretically present in all identified proteins when analyzing the amino acid composition. The oxidation-mediated Cys modifications in MV-treated samples were identified by comparing the predicted mass list of *in silico* trypsin-digested and biotinylated peptides (**Table 4**). This approach allowed us to confirm single peptides of RubisCO LSU, myrosinase, NAD(P)-binding Rossmann-fold-containing protein and FNR.

**Table 4:** Cysteines modified upon methyl viologen-mediated oxidation.

No.	Protein name	Peptide sequence	Predicted mass		Observed mass in MV treatment
			NEM	Biotin maleimide	
1	RubisCO large subunit	AVYEC*LR	978.0	1304.5	1304.61*
2	Myrosinase	C*SPKIDVR	1113.5	1440.0	1439.69*
19	NAD(P)-binding Rossmann-fold-containing protein	SLVSDSTSICGPSKFTGK	1938.9	2265.4	1938.1
21	Ferredoxin-NADP(+) oxidoreductase 1 (FNR1)	C*LLNTK	815.9	1142.4	1142.57*

The table shows the peptide sequence and the predicted mass in the presence of the alkylating agents either NEM or biotin maleimide. The mass observed in MALDI-TOF MS of the MV-treated sample is shown. NEM-labeled cysteine (C) mass matches to experimental mass value suggesting that these Cys are not modified during oxidation. C* denotes Cys in the peptide sequence which is labeled with biotin maleimide, since the predicted mass matched to the experimentally observed value.The underlined peptides were addressed to be modified also in *in vitro* oxidation treatment (refer ***Table 2***).

## DISCUSSION

In order to identify stroma protein targets sensitive to oxidation by H_2_O_2_, this work adopted a strategy similar to the “biotin switch”-method used to detect post-translational *S*-nitrosylation or glutathionylation (Jaffrey and Snyder, 2001; Lind et al., 2002). The present study relies on differential labeling of reduced thiols and formerly oxidized thiols, using two different alkylation reagents (NEM and biotin maleimide) of distinct molecular mass, which eased the preferential identification of the H_2_O_2_-sensitive proteins. Often redox proteomic studies identify redox-sensitive proteins by direct labeling of proteins during cell extraction, possibly leading to Cys oxidation during the lysis and labeling steps causing false positive results. The cross contamination assays indicate that the isolated chloroplast fractions were highly pure since the mitochondrial protein AtPrxII F was below detection limit (**Figure 1A**) and the cytosolic UGPase constituted less than 6% on a protein basis (**Figure 1B**). In the *in vitro* part of this work, the stroma proteins were completely reduced and then oxidized with low amounts of H_2_O_2_ to isolate only the most oxidant-sensitive protein thiols. It has been suggested that about 4 μmol H_2_O_2_ m^-2 ^leaf area^-1^ is formed in the chloroplast during photosynthesis under normal conditions (Foyer and Noctor, 2003). This value corresponds to about 300 μmol H_2_O_2_ L^-1^ stroma produced every second, assuming a leaf chlorophyll content of 300 mg^.^m^-2^ and a stroma volume of 40 μL mg^-1^ chlorophyll. The H_2_O_2_-detoxification capacity of the chloroplast ascorbate- and peroxiredoxin-dependent water-water cycles is high, and models predict that resting H_2_O_2_ concentrations are low as long as reductants are available (Polle, 2001). Here a low H_2_O_2_ concentration of about 10 μM equivalent to 10% of total protein thiols was used to identify primary targets of oxidation and can be considered to represent physiologically relevant conditions. In previous studies much higher concentrations of oxidants ranging from 1 mM up to 10 mM in non-stoichiometric amounts have been used in plant and animal redox proteomic studies. The results from such studies should be considered with care, since the employed concentrations are often outside the reasonable physiological range (Kim et al., 2000; Baty et al., 2002; Marx et al., 2003; Sethuraman et al., 2004; Winger et al., 2007).

The degree of labeling with biotin-maleimide increased in the H_2_O_2_-treated sample indicating the occurrence of oxidation-mediated Cys modification in our experiments (**Figure 3A**). Two-dimensional-immunoblots provided insight into the response of the H_2_O_2_-mediated oxidative changes. Linking our approach with MALDI-TOF analysis enabled the identification of proteins which are most sensitive to oxidation. Most proteins identified possess oxidation-susceptible Cys and are known to undergo dithiol-disulfide exchange reactions as reported in previous studies (Meyer et al., 2005; Ströher and Dietz, 2008; Lindahl and Kieselbach, 2009). All the identified proteins had functional annotations. Based on their function they can be generally categorized as enzymes involved in carbohydrate metabolism, photosynthesis, redox homeostasis, and nitrogen assimilation (**Table 1**).

### FUNCTIONAL CLASSIFICATION OF IDENTIFIED PROTEINS

**Figure A1:**
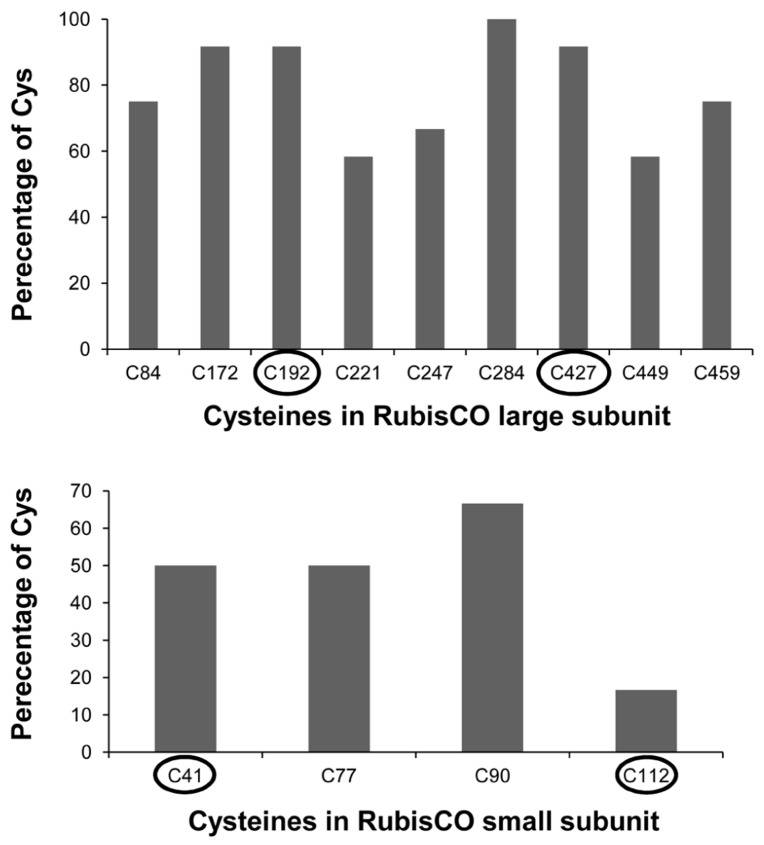
**Percentage of cysteine conservation in *A. thaliana* RubisCO large and small subunit protein in comparison to the following organisms: Rice, maize, Physcomitrella, Chlamydomonas, Chlorella, Euglena, Synechocystis, Nostoc, Hydrogenovibrio, *R. capsulatus* and *R. sphaeroides***. Encircled are the identified redox sensitive cysteines.

Enzymes such as phosphoribulokinase, GAPDH, and transketolase were identified as primary targets responding to reduction or oxidation of regulatory thiols. They were previously reported to be redox-regulated targets of Trx (Motohashi et al., 2001; Marchand et al., 2004). RubisCO was identified as one of the primary targets of oxidation. RubisCO plays a major role in photosynthesis, hence it is regulated by several mechanisms and redox-dependent modulation is one of them. Based on our MALDI-MS data analysis (**Table 2**) two Cys of RubisCO LSU C192 and C427 are predicted to be oxidized. In *Chlamydomonas reinhardtii* C192 was previously shown to be inactivated by arsenite (Moreno and Spreitzer, 1999). However, site-directed mutagenesis suggested that C192 lacks a role in disulphide-mediated inactivation. Rather C449 and 459 are assumed to be involved in redox-dependent catalytic inactivation and to trigger increased proteolysis of the protein (Moreno et al., 2008). In this study the identified C427 is conserved in 91% of the analyzed photosynthetic organisms (**Figure A1** in Appendix). In three-dimensional structures C427, 449, and 459 are in close proximity with basic rich amino acids, which could be essential for oxidative activity of the protein. Due to the high protein abundance in the millimolar range, RubisCO can be assumed to act as redox buffer to maintain a suitable redox state of the chloroplast in the presence of transiently increased ROS release. RubisCO SSU was identified as thioredoxin target by several studies (Motohashi et al., 2001; Balmer et al., 2003). In this work C41 and 117 were found to be modified by biotin maleimide due to oxidation, although the distance between both Cys is too far from each other (11–25 Å) to form a disulfide bond (Taylor and Andersson, 1997). But these Cys might be a target for SOH oxidation or mixed-disulfide formation and function as oxidant sensor. In *Chlamydomonas*, Zaffagnini et al. (2012) recently reported 225 glutathionylated proteins, among them many Calvin cycle enzymes, indicating a role of glutathionylation in protecting and regulating chloroplast carbohydrate metabolism. We can exclude glutathionylation, since the proteins were desalted following the reduction with DTT (see Materials and Methods). This step also eliminated any glutathione from the extracts.

In higher plant chloroplasts, Fd-GOGAT is a major enzyme for glutamate synthesis, involved in the conversion of glutamine and 2-oxoglutarate to glutamate. Thioredoxin-mediated redox regulation of Fd-GOGAT was addressed *in* *vitro* by several studies (Lichter and Häberlein, 1998; Motohashi et al., 2001; Balmer et al., 2003). The amino acid sequence of the protein shows 24 Cys residues in the mature form, three of which were alkylated with NEM (**Table 2**). FNR catalyzes the electron transfer between ferredoxin and NADPH, producing reducing equivalents for chloroplast metabolism and thus represents a crucial enzyme for various pathways requiring reductants. The predicted mass of a peptide containing a Cys residue matched the measured mass value (±0.5 Da) suggesting, that this particular Cys is sensitive to oxidation (**Table 2**). GAPDH is subjected to post-translational modifications which involve thiol-disulfide transitions of regulatory Cys, complex formation with ribulose-5-phosphate kinase and a regulatory protein named CP12 (Scheibe et al., 2002). This mechanism allows the coordination of GAPDH redox regulation with availability of its substrate 1,3-bisphosphoglycerate.

Proof of concept is also provided by the identification of 2-Cys Prx and cylophilin 20-3. 2-Cys Prx is among the top 20 most abundant stroma proteins (Peltier et al., 2006), functions as high-affinity thiol-peroxidase (König et al., 2003), and undergoes large redox-dependent conformational changes linked to functional switches (Dietz, 2012). Here the recombinant 2-Cys Prx protein was a convincing system to test and validate the various steps of oxidation, blocking, labeling, and detection in the work flow (**Figure 2B**), and its recovery *in vitro* and *in vivo* shows also, that the early oxidizable proteins are indeed trapped with this method (**Tables 1–3**). Likewise cylophilin 20-3 is a known target of thiol/disulfide transition (Laxa et al., 2007) and this redox switch affects its peptidylprolyl-cis/trans isomerase activity and probably also its capability for protein/protein interactions.

### REDOX MODIFICATION OF PROTEINS UPON OXIDATIVE STRESS *IN VIVO*

The *in vivo* redox status of protein thiols was monitored using MV-mediated oxidative stress. The majority of the identified proteins are involved in antioxidant defense (**Table 3**). GST catalyzes hydroperoxide detoxification in the presence of glutathione. GST tau is also known to detoxify herbicides and plays a role in signal transduction (Neuefeind et al., 1997; Dixon et al., 2003). Thiol-mediated light/dark regulation of Calvin cycle enzymes is a well known process for years (Buchanan, 1980). In this line SBPase and fructose-bisphosphate aldolase were identified. SBPase is activated by disulfide reduction. The involved Cys have been identified by site-directed mutagenesis (Dunford et al., 1998). Interestingly overexpression of SBPase stimulates growth during early development and under stress suggesting that SBPase activity controls fluxes in the Calvin cycle to a major extent (Lefebvre et al., 2005; Feng et al., 2007). The sensitivity of SBPase to oxidation *in vivo* may provide an explanation why SBPase plays a particular role under stress with increased ROS production.

Regulators of PS II oxygen-evolving complex (OEC) such as PsbO-1, PsbP-1, and PsbQ were also identified as oxidation-sensitive proteins. A recent study has proposed the presence of an intra-molecular disulfide bond in PsbO-1 and PsbP-1 using a diagonal two-dimensional gel (Ströher and Dietz, 2008). PsbQ indeed has no Cys but could be associated and coeluted with other PS II OEC proteins. Biochemical studies showed that *A. thaliana* nucleoside diphosphate kinase 2 (NDPK2) is associated with MAPK-mediated H_2_O_2_ signaling in plants (Moon et al., 2003). Being one of the early oxidation targets, NDPK2 thiols might play a major role in the ROS-mediated signaling pathway. The *in vivo *experimental strategy should be improved in the future, e.g., by partial removal of RubisCO, followed by enrichment of less abundant proteins. The here applied method does not distinguish between direct oxidation by H_2_O_2_ and proximity-based oxidation mechanism where thiol proteins such as peroxiredoxins first react with H_2_O_2_ to form a SOH intermediate, which then oxidizes a thiol of another protein (König et al., 2012).

## CONCLUSION

In conclusion, the here developed approach provides insight into early targets of oxidation by H_2_O_2_ within the stroma proteome under physiologically relevant conditions. This method is applicable to identify redox-dependent protein modifications *in vitro* and *ex vivo*, e.g., redox changes upon exogenous MV treatment but also in response to other stresses. The study identified expected early targets such as 2-Cys peroxiredoxin which has been classified as redox sensor and many known targets of redox regulation such as SBPase and FNR. Redox input elements, transmitters, targets, and sensors form the cellular redox regulatory network (Dietz, 2008). This network needs to be expanded by the functional element redox buffer proteins. RubisCO is composed of eight LSU and eight SSU. LSU contains 9, SSU 4-Cys residues in *A. thaliana*. With a concentration of about 500 μM RubisCO, the RubisCO thiols represent the largest thiol pool of the stroma that exceeds any other chloroplast compound (Jacquot et al., 2013). A future challenge will be to monitor the diverse thiol modifications such as S-nitrosylation, glutathionylation, and intra- or interpeptide disulfide formation in parallel and to dissect the spatial and functional specificity of these modifications under conditions of environmental changes.

## Conflict of Interest Statement

The authors declare that the research was conducted in the absence of any commercial or financial relationships that could be construed as a potential conflict of interest.

## Abbreviations

Cys: cysteine
2-Cys Prx: 2-cysteine peroxiredoxin
2DE: two-dimensional gel electrophoresis
DTNB: 5,5-dithio-bis(2-nitrobenzoic acid)
DTT: dithiothreitol
GAPDH: glyceraldehyde-3-phosphate dehydrogenase
HCF: high chlorophyll fluorescence
MALDI-TOF: matrix-assisted laser desorption/ionization-time of flight
NEM: *N*-ethylmaleimide
ROS: reactive oxygen species
RubisCO: ribulose-1,5-bisphosphate carboxylase/oxygenase
TCA: trichloroacetic acid.
